# Expert recommendations for the care of newborns of mothers with COVID-19

**DOI:** 10.6061/clinics/2020/e1932

**Published:** 2020-05-11

**Authors:** Werther Brunow de Carvalho, Maria Augusta Bento Cicaroni Gibelli, Vera Lucia Jornada Krebs, Valdenise Martins Laurindo Tuma Calil, Cíntia Johnston

**Affiliations:** I Terapia Intensiva em Neonatologia/Pediatria, Departamento de Pediatria, Faculdade de Medicina (FMUSP), Universidade de Sao Paulo, Sao Paulo, SP, BR; II Centro Neonatal e Terapia Intensiva Neonatal, Instituto da Crianca e do Adolescente (ICr), Hospital das Clinicas HCFMUSP, Faculdade de Medicina, Universidade de Sao Paulo, Sao Paulo, SP, BR; III Curso de Pos-Graduacao, Departamento de Pediatria, Faculdade de Medicina (FMUSP), Universidade de Sao Paulo, Sao Paulo, SP, BR

**Keywords:** Pregnant Women, Newborn, COVID-19 infection, NICU, Guideline

## Abstract

This article presents expert recommendations for assisting newborn children of mothers with suspected or diagnosed coronavirus disease 2019 (COVID-19). The consensus was developed by five experts with an average of 20 years of experience in neonatal intensive care working at a reference university hospital in Brazil for the care of pregnant women and newborns with suspected or confirmed COVID-19.

Despite the lack of scientific evidence regarding the potential for viral transmission to their fetus in pregnant mothers diagnosed with or suspected of COVID-19, it is important to elaborate the lines of care by specialists from hospitals caring for suspected and confirmed COVID-19 cases to guide multidisciplinary teams and families diagnosed with the disease or involved in the care of pregnant women and newborns in this context. Multidisciplinary teams must be attentive to the signs and symptoms of COVID-19 so that decision-making is oriented and assertive for the management of the mother and newborn in both the hospital setting and at hospital discharge.

## INTRODUCTION

To date, the impact of coronavirus disease 2019 (COVID-19), the disease caused by infection by severe acute respiratory syndrome coronavirus 2 (SARS-CoV-2) during pregnancy is not fully known. Although there remains no evidence of transplacental transmission of the virus, this possibility may exist 1-7. In this context, it is necessary to determine the likely mechanisms of contamination of the fetus, such as maternal fluids and intrapartum, as well as neonatal factors that may influence the perinatal transmission of the virus 8.

The clinical signs and symptoms, results of laboratory tests, and chest tomography findings of nine pregnant women with COVID-19 pneumonia in Wuhan-China were recently described 9. These women were diagnosed based on oral swabs positive for SARS-CoV-2. Vertical intrauterine transmission was investigated by testing amniotic fluid, cord blood, and oral swabs from the newborns as well as breast milk, all of which were negative for the presence of the virus in all mothers and their newborns.

Premature birth occurs in an estimated 47% of pregnant women with confirmed COVID-19. Among 19 neonates of mothers positive for COVID-19, delivery occurred up to 13 days after disease onset. In newborns born to mothers with COVID-19, the clinical findings ranged from asymptomatic to manifestations such as respiratory distress requiring ventilatory support, disseminated intravascular coagulation, multiple organ dysfunction, and shock. Despite the presence of signs and symptoms in the mothers, these newborns were negative for COVID-19 4.

Fan et al. 5 reported two cases of COVID-19 in the third trimester of pregnancy. The mothers and their newborns showed good clinical evolution. The virus was not detected in breast milk or in swabs or serological samples from the newborns. The authors concluded that the risk of infection by vertical transmission of SARS-CoV-2 was low. Another case report 10 presented a preterm newborn (gestational age 30 weeks) whose mother was symptomatic with a positive swab for COVID-19 2 days before delivery. Oral swabs and feces from the newborn tested negative for coronavirus on the third, seventh, and ninth days after birth.

Hong et al. 10 proposed the presence of at least one of following clinical signs or symptoms as criteria for the neonatal diagnosis of COVID-2: thermal instability, hypoactivity, feeding difficulty, respiratory distress, chest X-ray with changes (including single or bilateral ground-glass patterns), COVID-19 diagnosis in family or caregiver of the newborn, intimate contact with people with suspected or confirmed COVID-19, or patients with unclear pneumonia.

Based on the reports in the literature so far on the subject, as well as on the expertise of the authors, the objective of this article was to present expert recommendations for assisting the newborn children of mothers with suspected or diagnosed COVID-19.

## RECOMMENDATIONS FOR NEONATAL CARE

### Personal Protective Equipment (PPE) and Insulation Precautions

SARS-CoV-2 is a respiratory virus transmitted person-to-person mainly by respiratory droplets. The infection is mediated by virus present in the respiratory secretions of an infected person that come into contact with the mucous membranes of another person. High-risk exposure is defined as a person with COVID-19 disease requiring direct physical or close contact (<1.8 meters) for an extended time.

It is recommended that precautions be taken to prevent droplets and contact, during contact between newborns and their mothers with COVID-19 through the use of PPE including aprons, gloves, surgical masks, and eye protection (goggles or face protector) 8,.

Contact and droplet precautions are recommended in situations that could generate aerosols; these precautions include the use of PPE such as aprons, gloves, N95 or PFF2 masks, and eye protection (glasses or face protector). Consideration of the potential for generating aerosols is recommended, including bag-mask ventilation, tracheal intubation, airway aspiration, tracheal extubation, high-flow nasal oxygen therapy (oxygen flow greater than 2 liters per minute/kg), and non-invasive ventilation (continuous positive airway pressure [CPAP] or bilevel) 20,21.

### Assistance in the Delivery Room

Following the normal routine assistance for newborns is recommended in the delivery room according to the hospital. During newborn resuscitation, multidisciplinary teams must be fitted with PPE as a precaution against transmission by air, droplets, and contact due to the increased probability of aerosols with maternal viruses and the potential need for tracheal intubation, airway aspiration, and ventilation with positive pressure, which can also generate aerosols for the newborn.

### Recommendations for the Care of Pregnant Women

- Collect COVID-19 samples from pregnant women in labor with suspected COVID-19 or with disease confirmation in the 14 days preceding labor.
- Suspend skin-to-skin contact between the mother and newborn in cases of symptomatic parturients or those with home contact with a person with flu-like symptoms or positive for COVID-19.
- After the birth, refer the puerperal woman to the inpatient sector for the suspected and confirmed patients of COVID-19.

### Recommendations for Newborn Care

- Newborns should be resuscitated under radiant heat in the delivery room.
- The multidisciplinary team in contact with the newborn must wear PPE (N95 or PFF2 masks, glasses and face protectors, waterproof aprons, and sterile gloves and caps).
- In cases of newborns with respiratory distress, treatment must follow the assistance routines of each service.

### Newborn Transport and ICU Admission

It is recommended that all newborns of pregnant women in labor with suspicion or confirmed COVID-19 in the 14 days preceding labor be transported in a heated incubator from the delivery room to the neonatal center or neonatal intensive care unit (ICU) and admitted to an isolation room. The following steps are recommended for the dressing and undressing of the transport team:

- Wear all garments until arrival at the neonatal ICU.
- If individual rooms with negative pressure are available, the hospitalization of newborns positive for COVID-19 should be prioritized.
- Remove gloves and aprons in the patient's room and caps, glasses, and masks in the anteroom.

Newborns requiring intensive care should ideally be admitted to an isolation room with the potential for negative ambient pressure or other available air filtration systems. If this feature is not available, cribs or incubators should be kept at a minimum distance of 1.5 meters. For newborns with indications for non-invasive ventilation (CPAP or bilevel), high-flow oxygen therapy, or invasive mechanical ventilation, precautions should be taken to avoid aerosols, droplets, and contact thought the use of PPEs recommended for this purpose.

### Care in the Neonatal Center and Clinical Evaluation of Newborns

The following routine care is recommended for newborns upon arrival to the Neonatal Center based on the infant’s clinical condition and COVID-19 severity:

a) Asymptomatic newborns and mothers negative for COVID-19

- If the puerperal woman is clinically well, she can receive the newborn in the Joint Accommodation (JA); at discharge, they should be referred to the basic health unit to receive routine guidance.

b) Asymptomatic newborns awaiting the mother's COVID-19 test results

- The newborn must be kept in the isolation sector to prevent contact and droplets and should be bathed immediately after birth to remove viruses potentially present on the skin surface.
- The multidisciplinary team must use precautions against droplets and close contact.
- The mother will be able to receive the newborn at the JA after being instructed on cough etiquette, hand hygiene, use of a surgical mask for newborn routines and breastfeeding, maintaining a distance of at least 1.8 meters between the newborn's crib and the postpartum bed. Breastfeeding should be started as soon as possible after hygiene care and measures to prevent contamination of the newborn.
- The maintenance of a single (regular) companion is recommended, provided that he or she is asymptomatic and does not have home contact with a person with flu-like symptoms or positive for COVID-19, in places that promote distance between hospitalized patients or with private accommodation.
- Routine newborn care: 1^st^ day: vaccination against hepatitis B and vitamin K injection; 2^nd^ day: red reflex and otoacoustic emission, tongue; pulse oximetry, and neonatal screening tests with 48 hours of birth.
- For mothers positive for COVID-19: the indications for testing of asymptomatic newborns are evaluated on a case-by-case analysis.
- At hospital discharge, the person responsible for the newborn should receive guidance on home isolation care and warning signs. Social services must provide a support network including a family member or guardian (wearing a surgical mask) at the time of discharge who can provide home newborn care for mothers under investigation or positive for COVID-19.

c) Symptomatic newborns

Recommendations for the care of newborns with acute ventilatory failure:

- Intern in an isolation room.
- COVID-19 investigation.
- Among diagnostic hypotheses, consider COVID-19 infection.
- Start treatment according to the service's protocol.
- Multidisciplinary team wearing PPE to protect against aerosols and contact.
- Deparamentation: removal of gloves and apron in the patient's room and removal of caps, glasses, and mask in that order in the anteroom before entering the hall.

The risk of postnatal infection in the immediate postpartum period due to mother-newborn contact has not yet been established. Temporary separation of the mother from her newborn will minimize the risk of postnatal infection from maternal respiratory secretions. The likely benefits of this temporary separation to decrease the risk of neonatal infection should be discussed with the mother, preferably before delivery.

### Breastfeeding

The presence of coronavirus in breast milk has not yet been demonstrated. Mothers can express breast milk (after hand and breast hygiene), which can be offered to the newborn by caregivers. Pumps and accessories must be thoroughly cleaned according to the standard measures of the breast milk banks, which must include cleaning the pump with disinfectant wipes and washing the accessories with hot water and soap. In addition to the known benefits of breastfeeding, mothers' milk can provide infant protection factors after maternal SARS-CoV-2 infection.

### Viral Testing of the Newborn

If available, molecular tests for SARS-CoV-2 infection should also be performed in newborns. This strategy can facilitate care planning after hospital discharge and contribute to the understanding of viral transmission. If the test is not readily available or there is a shortage, clinicians may choose to perform only clinical monitoring. Newborns requiring care in the intensive care unit should be tested to determine the potential contribution of COVID-19 to the observed clinical disease. The testing of newborns in the intensive care unit will allow scheduling of discontinuation of contact precautions (droplets and aerosols) for negative results.

The need for testing and the ideal time for testing has not yet been established. Viral detection data are limited. The following are recommended to distinguish transient viral colonization from established infection:

- The first molecular test must be carried out within approximately 24 hours of birth.
- The test should be repeated with approximately 48 hours after birth. For newborns in good general condition who will be discharged within 48 hours of birth, this test may not be performed. However, the possibility of newborns with negative results within 24 hours of birth becoming positive within 48 to 72 hours of birth cannot be ruled out.
- Newborns requiring hospitalization can be transitioned to the use of precautions (contact/droplets/aerosols) following two negative test results at least 24 hours apart. For neonates with positive initial CRP test results, follow-up tests should be performed at intervals of 48 to 72 hours until two consecutive negative results are obtained.

### Maternal and Family Visits to Hospitalized Newborns

Symptomatic mothers or fathers or those who have had home contact with individuals with influenza syndrome or positive for COVID-19 should not visit hospitalized newborns until they become asymptomatic and the period of COVID-19 transmissibility has passed (approximately 14 days).

Daily screening for respiratory symptoms and flu syndrome is recommended for fathers and mothers visiting newborns. In cases in which the father or mother cannot stay, the family may designate an asymptomatic substitute without home contact with an individual with flu syndrome or positive for COVID-19.

### Newborn Hospital Discharge

Newborns should be discharged according to the criteria of each hospital and avoiding the use of public transport. Social visits at home to the mother and newborn should be suspended. The following situations are recommended to be considered:

- Asymptomatic newborn with no results or no tests for COVID-19: can be discharged home with due precautions and outpatient monitoring by phone or telemedicine for up to 14 days after birth. Guidance on the use of gloves and hand hygiene should be provided to family members and caregivers. Uninfected individuals over 60 years of age and with comorbidities should not care for the newborn.
- Newborns with negative molecular tests for COVID should be discharged from the care of an uninfected caregiver. If the mother is in the same house, she should keep a distance of at least 1.8 m for as long as possible. When the mother is closest to the newborn, she should wear a surgical or tissue mask and frequently perform hand hygiene. The distance between the mother and the newborn must be maintained until negative COVID-19 results are obtained for at least two consecutive nasopharyngeal swab samples collected at an interval of ≥24 hours.
- Caregivers under observation for the development of COVID-19 should wear masks and perform hand hygiene until their test results are known.
- Frequent outpatient follow-up by telephone or telemedicine or face-to-face assessments up to 14 days after hospital discharge are recommended for newborns positive for COVID-19 or at risk of postnatal coronavirus infection who are unable to be tested.

At the time of hospital discharge, mothers should be educated on the warning signs of illness in her newborn; namely:

1. Fever;
2. Poor feeding or vomiting or bloating;
3. Lethargy, drowsiness;
4. Breathing difficulty, groaning;
5. Worsening jaundice intensity;
6. Decreased diuresis;
7. Pallor;
8. Cyanosis.

In the presence of any of the above clinical signs, the following are recommended:

- Identify the Health facility closest to the residence.
- If positive for COVID-19: use a mask and inform the health professional who provides care to the family.
- If the mother still does not have her COVID-19 screening results, advise her to contact the hospital by phone.

### Recommendations for home isolation of mothers positive or under investigation for COVID-19

- Maintain breastfeeding as long as the mother desires and is in adequate condition;
- Educate the mother regarding precautions for the prevention of virus transmission (contact/droplets/aerosols) during contact with the infant, including breastfeeding, such as: washing hands for at least 20 seconds before touching the newborn and before removing breastmilk (by hand or pump); wearing a face mask (completely covering the nose and mouth) while breastfeeding; avoiding talking or coughing while breastfeeding; and changing the mask immediately in case of coughing or sneezing or with each breastfeeding;
- Strictly follow the recommendations for cleaning breast pumps after each use.
- Consideration should be given to requesting the help of a person without evidence of viral infection to offer the newborn breast milk in a cup, cup, or spoon (this person must learn how to do this task with help from a health professional).

## CONCLUSIONS

Despite the lack of scientific evidence regarding the potential viral transmission to their fetus by pregnant women with suspected or positive for COVID-19, multidisciplinary teams must be attentive to the disease signs and symptoms for guided and assertive decision making in the management of both mothers and newborns in the hospital environment and discharge.

## AUTHOR CONTRIBUTIONS

Carvalho WB, Gibelli MABC, Krebs VLJ, Calil VMLT and Johnston C were responsible for the study conception, data curation, formal analysis, funding acquisition, investigation, methodology, project administration, software resources, study supervision, validation, manuscript drafting, editing and review.
